# Impact of Agro-Industrial Side-Streams on Sesquiterpene Production by Submerged Cultured *Cerrena unicolor*

**DOI:** 10.3390/foods12030668

**Published:** 2023-02-03

**Authors:** Nils Püth, Franziska Ersoy, Ralf G. Berger, Ulrich Krings

**Affiliations:** Institute of Food Chemistry, Leibniz University Hannover, Callinstraße 5, 30167 Hannover, Germany

**Keywords:** Basidiomycota, *Cerrena unicolor*, medium supplementation, rapeseed press cake, side-streams, sesquiterpene synthesis, bioeconomy

## Abstract

The quality and harvest of essential oils depend on a large number of factors, most of which are hard to control in an open-field environment. Therefore, Basidiomycota have gained attention as a source for biotechnologically produced terpenoids. The basidiomycete *Cerrena unicolor* (Cun) was cultivated in submerged culture, and the production of sesquiterpenoids was analyzed *via* stir bar sorptive extraction (SBSE), followed by thermo-desorption gas chromatography coupled with mass spectrometry (TDS-GC-MS). Identification of aroma-active sesquiterpenoids was supported by GC, coupled with an olfactory detection port (ODP). Following the ideal of a circular bioeconomy, Cun was submerged (up-scalable) cultivated, and supplemented with a variety of food industrial side-streams. The effects of the different supplementations and of pure fatty acids were evaluated by liquid extraction and analysis of the terpenoids *via* GC-MS. As sesquiterpenoid production was enhanced by the most by lipid-rich side-streams, a cultivation with ^13^C-labeled acetate was conducted. Data confirmed that lipid-rich side-streams enhanced the sesquiterpene production through an increased acetyl-CoA pool.

## 1. Introduction

Fungi are one of the most versatile producers of secondary metabolites, including beneficial (antibiotics, pharmaceuticals, and perfumery) and harmful (toxins and carcinogens). These are derived from four main chemical classes: polyketides, non-ribosomal peptides, indole alkaloids, and terpenic compounds [1]. Basidiomycota produce a large number of different sesquiterpenes, whose biological functions encompass physiological effects, defense against other organisms (especially parasites), attraction of pollinators, and phytohormone activity [2,3,4,5].

Fungal volatile terpenes, formerly thought to occur in plants only, were first reported almost 60 years ago [6]. Since then, especially higher fungi, such as basidiomycetes (mushrooms), have been identified as a good source of aroma compounds. Their volatile profile consists of aliphatics, aromatics, and terpenoids, whose complexity can be comparable to the complexity of the plant volatilome [7]. Expressive examples for aroma-active sesquiterpenoids are the oxo-functionalized, grapefruit-smelling (+)-nootkatone and the fruity smelling *α*-ylangene [8,9]. The sustainable biotechnological production of sesquiterpenoids is not only of interest for the production of aromas but also for pharmaceuticals [10], due to their anti-bacterial, anti-inflammatory, anti-oxidative, and cytotoxic effects [11,12,13,14,15,16].

*Cerrena unicolor* (Cun) is a white-rot basidiomycete and a known producer of several bioactive compounds [17,18]. The fungus is also well-known as a source of miscellaneous enzymes, such as laccases, xylanases, cellulases, oxidases, and, as was recently reported, terpene cyclases [19,20,21,22,23]. In 2018, Cun was discovered to be a producer of volatile aroma-active compounds [24]. Its fruity, flowery scent with woody notes aroused interest for further investigations. Sesquiterpenoids were identified as aroma-active principles built by this fungus.

Fungal sesquiterpene biosynthesis starts with acetyl-coenzyme A (acetyl-CoA) to form the two C5-terpene building blocks, isopentyl and dimethylallyl pyrophosphate (IPP and DMAPP), along the well-known mevalonate pathway. Three building blocks result in the characteristic farnesyl backbone of sesquiterpenes, consisting of 15 carbon atoms. The linear farnesyl pyrophosphate (FPP) serves as the substrate for sesquiterpene synthases, which synthesize specific cyclic sesquiterpenes [25]. After the metal ion-induced release of the pyrophosphate group, the resulting highly reactive allylic cation is stabilized in the active center of a respective cyclase. A subsequent cascade of rearrangements, hydride shifts, and formation of C–C bonds (regiospecific cyclisation) furnishes sesquiterpene and later, the oxidized sesquiterpenoid products [26]. A recent study showed that the synthesis of sesquiterpenes was associated with the sporulation process in fruiting bodies [27]. However, Cun also produced sesquiterpenes in submerged cultures, thus enabling the set-up of an experimental model system for further scaling-up.

Several chemical and physical factors may affect the production of sesquiterpenoids in submerged, cultured basidiomycetes; however, scarce information was found in literature [28]. The use and valorization of food and agro-industrial side-streams as cultivation substrates may render fungal bioprocesses more sustainable [29,30,31], as these fungi thrive well on such complex substrates. In this work, a variety of side-streams of different chemical compositions were used as supplements for the submerged cultivation of Cun. Their impact on culture growth and aroma-active terpene formation was evaluated to identify the preferred side-stream. Protein-, lipid-, and carbohydrate-rich side-streams were selected, and the mevalonate pathway towards sesquiterpene synthesis was eventually confirmed using ^13^C-labeled acetate.

## 2. Materials and Methods

### 2.1. Chemicals

All chemicals, solvents, and medium components were purchased from Carl Roth (Karlsruhe, Germany), VWR (Radnor, PA, USA), Sigma Aldrich (St. Louis, MO, USA), and Merck KGaA (Darmstadt, Germany) unless mentioned otherwise. All solvents were purified by rectification prior to use. Side-streams were obtained from: rapeseed press cake from Teutoburger Ölmühle (Ibbenbüren, Germany), maple wood chips from Axtschlag GmbH (Heidesee, Germany), wheat bran from DM (Karlsruhe, Germany), grape pomace from Staatsweingut Neustadt (Neustadt a.d.W., Germany), and lemon peel from H. Zorn (JLU Gießen, Germany). Rapeseed oil was purchased from Rapso (Aschach, Austria) and coconut butter from Rewe (Köln, Germany).

### 2.2. Cultivation Conditions

All flasks and media were autoclaved prior to use. For submerged cultivation, standard nutrient liquid (SNL) at pH 6.0 (30 g/L D-glucose monohydrate, 4.5 g/L L-asparagine monohydrate, 3 g/L yeast extract, 1.5 g/L KH_2_PO_4_, 0.5 g/L MgSO_4_, 5 µg/L CuSO_4_ × 5 H_2_O, 80 µg/L FeCl_3_ × 6 H_2_O, 30 µg/mL MnSO_4_ × H_2_0, 90 µg/mL ZnSO_4_ × 7 H_2_O, 400 µg/mL EDTA) was used. 

Pre-cultures of 250 mL were inoculated with a 1 cm^2^ grown agar plate and homogenized with a disperser (Miccra D-9, Miccra GmbH, Heitersheim). Main cultures of 250 mL were inoculated with 10% (*v*/*v*) pre-culture. Cultivations were performed in triplicates. As side-streams, wheat bran, potato starch, maple wood chips, rapeseed press cake, grape pomace, and lemon peel (2% *w*/*v*) were added to the main culture upon inoculation. As lipidic supplements, rapeseed oil, linoleic acid, oleic acid, and coconut butter (2% *v*/*v*) were used.

### 2.3. Extraction of Volatiles

For endpoint product analysis, volatiles were extracted from 250 mL of culture supernatant with an azeotropic mixture of *n*-pentane and diethyl ether (PE; 1:1.12 *v*/*v*). Samples were dried with sodium sulfate and concentrated *via* Vigreux rectification (DURAN, Wertheim, Germany) to a final volume of approximately 1 mL. Analysis was performed *via* thermodesorption–gas chromatography–mass spectrometry and olfactometry (TDS-GC-MS/O), as described below (*cf.* 2.5) [32]. Semi-quantification was performed using an internal standard (methyl dodecanoate), with a final concentration of 50 µg/mL.

For time-dependent product analysis, culture supernatant was extracted *via* sequential stir bar sorptive extraction (SBSE) using polydimethylsiloxane-coated Twisters (10 mm × 0.5 mm; Gerstel, Mülheim, Germany) [33]. Extraction was performed with 5 mL of culture supernatant for one hour under vigorous stirring at 400 rpm. The Twister was rinsed with deionized water, dried with a lint-free cloth, and stored at 4 °C. A second extraction was performed by adding a new Twister and NaCl with a final concentration of 30% (m/v) to the supernatant. Both Twisters were analyzed together *via* gas chromatography.

### 2.4. GOPOD-Assay, Determination of D-Glucose Concentration

D-Glucose was analyzed using a glucose oxidase/peroxidase assay (GOPOD) by Megazyme (Bray, Ireland) according to the manufacturer’s protocol. External calibration was performed with 0.05–1.0 mg/mL D-Glucose. Determinations were performed in duplicates.

### 2.5. GC-MS Analysis

The system consisted of an Agilent 6890N GC (Agilent, Santa Clara, CA, USA) and an Agilent 5975B MS (interface: 300 °C, ion source: 200 °C, quadrupole: 100 °C, electron impact ionization: 70 eV, scan range: 33–300 *m*/*z*). Stationary-phase DB-Wax (30 m, 0.25 mm, 0.25 µm) and DB-5ms (30 m, 0.25 mm, 0.25 µm) from Agilent Technologies were used. Injection was performed *via* a thermal desorption (TDS-3) and cold injection system (CIS-4) with an autosampler TDSA-2 (Gerstel, Mülheim an der Ruhr, Germany). Helium with a flow rate of 1.3 mL/min was used as the mobile phase. The temperature program was 10 min of hold-time at 40 °C, 5 °C/min until 250 °C, and 10 min hold-time at 250 °C. Compound identification was performed *via* retention index (polar/non-polar), comparison of the MS spectra (NIST, Wiley spectral database), and when possible, *via* authentic standard and odor impressions.

### 2.6. Lipid Content Determination

A defined amount of side-stream was applied to a Soxhlet shell and extracted with 200 mL of pentane/diethyl ether in a B-811 extraction system (BÜCHI Labortechnik AG, Flawil, Switzerland). After 25 cycles, the solvent was evaporated and the beaker weighed to constant mass. Extractions were performed in duplicates.

### 2.7. Fatty Acids Profiling

Approximately 100 mg of oil from the lipid content determination was solved in 1 mL of hexane. Sodium methylate was added to a final concentration of 1%, and the mixture was incubated at 60 °C and 200 rpm for 30 min. The organic phase was dried with sodium sulphate and diluted tenfold with hexane. The mixture was analyzed *via* TDS-GC-MS, as reported above *(cf*. 2.5). Determinations were performed in duplicates.

### 2.8. Protein Content Determination

The determination of the protein content of side-streams was performed with a K-424 digestion unit and a K-350 distillation unit (BÜCHI Labortechnik AG, Flawil, Switzerland) according to the manual. Determinations were performed in duplicates.

## 3. Results and Discussion

In preliminary experiments, *Cerrena unicolor* (Cun) was identified as a potent producer of sesquiterpenes and other aroma compounds [24]. Submerged cultures of the fungus provided a rich, fruity–flowery volatilome, including various sesquiterpenes and sesquiterpenoids (Table 1). Nine days after inoculation in SNL medium, D-glucose was fully metabolized, and an increased formation of volatiles started.

Volatiles extracted from the culture broth were tentatively identified according to their linear retention indices on two stationary phases of different polarity, electron impact (EI) mass spectra, and, if available, using authentic standards. Extracted ion chromatograms (EICs) of *m*/*z* 204, 218, 220, and 222 were used for selective detection and semi-quantitation of sesquiterpenes and respective terpenoids (alcohols, ketones, and aldehydes). In total, 13 cyclic sesquiterpene hydrocarbons, alcohols, and aldehydes were identified (Table 1). Based on the underlying cyclization mechanisms, sesquiterpene synthases can be grouped into four clades [34,35]. Formation of *α*-muurolene, *α*-copaene, *β*-cubebene, and epicubenol can be assigned to cyclases of clade I and II, which form sesquiterpenes along a 1,10-ring closure of an intermediate *trans*- or *cis*-farnesyl cation, respectively. However, *β*-curcumene and *α*-bisabolol are representative products of clade III cyclases that catalyze the 1,6-cyclization of farnesyl cations. Humulene is the only product associated with clade IV cyclases detected in the culture broth of Cun under the chosen conditions.

**Scheme 1:**
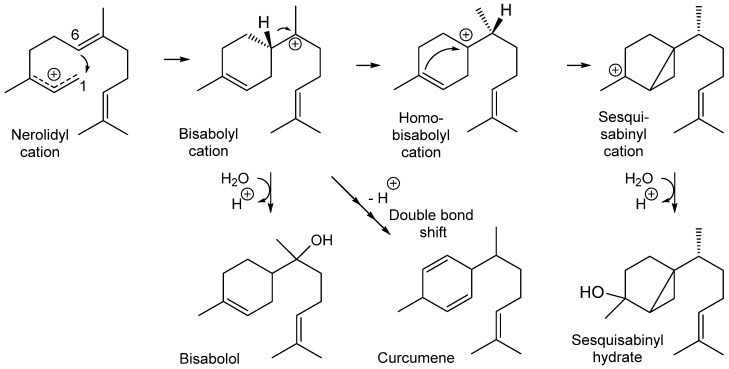
Proposed biogenetic relationship of bisabolol, curcumene, and sesquisabinene hydrate in the submerged, cultured of *Cerrena unicolor* (stereochemistry incomplete to facilitate reading).

Apart from the common 1,10 closure of (*E*,*E*)-farnesyl diphosphate, which resulted in the formation of cadinane and eudesmane skeletons, a 1,6 closure of nerolidyl pyrophosphate is supposed to yield the rather rare sesquisabinene and its hydrate. Their biogenesis is rationalized by a sequence starting with a nerolidyl cation, which, in few steps, is converted first to bisabolol, then to curcumene and sesquisabinene hydrate (Scheme 1), all found in the supernatant of Cun cultures (Table 1).

This hypothesis is supported by the co-occurrence of bisabolene and sesquisabinene skeletons, for example, in Indian sandalwood oil [36], indicating that the fungus and the taxonomically distant tree may follow similar pathways.

### 3.1. Formation Kinetics in Standard Nutrient Liquid Medium (SNL)

**Figure 1:**
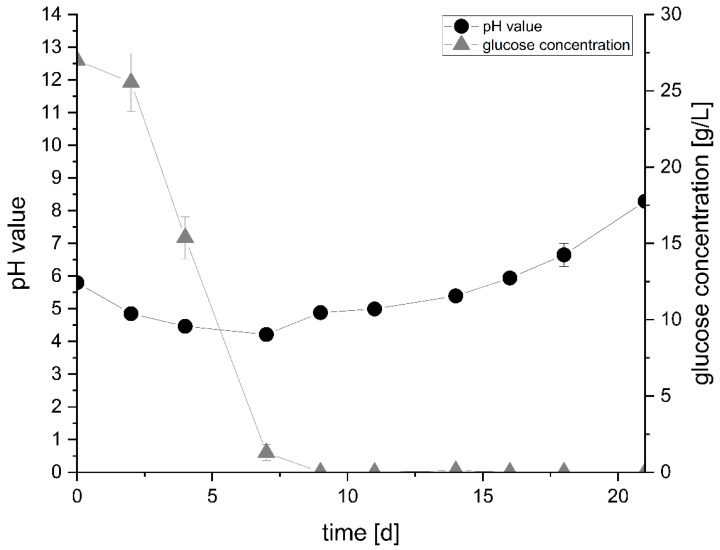
D-Glucose concentration and pH during the cultivation of *Cerrena unicolor* in SNL medium at 150 rpm and 24 °C for 21 days.

Product formation kinetics were monitored for 21 days in main cultures of Cun cultivated in 250 mL of SNL medium using sequential stir bar sorptive extraction. A sample of 7 mL was taken every two to three days and volatiles, pH, and D-glucose concentrations were determined (Figure 1).

The D-glucose concentration and the pH value over the course of cultivation are shown in Figure 1. Without a distinct lag phase, the D-glucose concentration in the medium declined rapidly, and D-glucose was completely exhausted within 9 days of cultivation. The pH value started in the slightly acidic range at pH 6 and decreased to pH 4.5 until day 7. With the depletion of the carbon source after 9 days, the pH value increased and reached pH 8.3 after 21 days.

**Figure 2:**
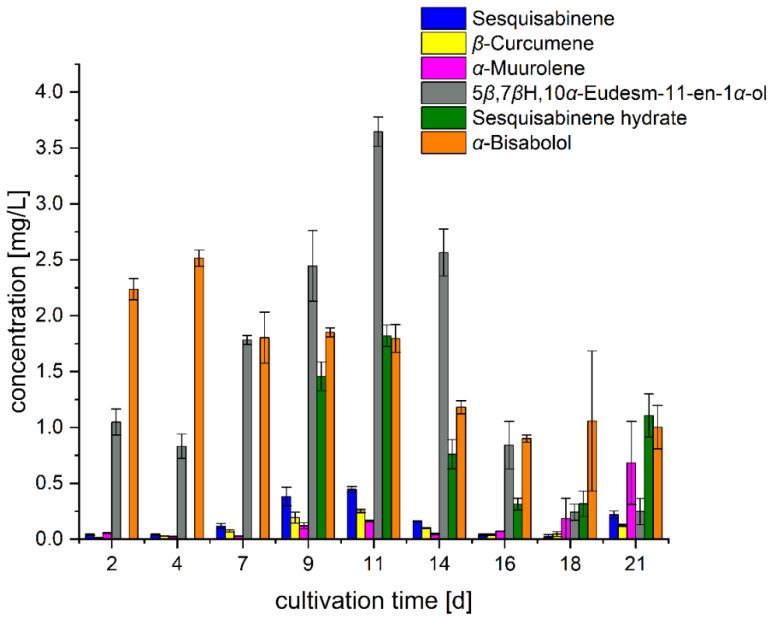
Sesquiterpenoid production over 21 days during the submerged cultivation of *Cerrena unicolor*.

The increasing pH value might indicate the switch from primary to secondary metabolism [1]. In literature, a pH shift is often associated with the start of secondary metabolism [37]. However, in this study, a clear distinction between primary and secondary metabolism could not be made, as sesquiterpene compounds were isolated from the cultivation medium right after the start (Figure 2). All of them were present from the beginning but in low concentrations (<2.5 mg/L), most likely due to a carry-over from the liquid pre-cultures used for inoculation. Upon D-glucose depletion after day 7, a moderate increase was observed for all products until culture day 11. Sesquiterpenoids are known to be produced during secondary metabolism [38,39,40]. However, after day 11, the product concentration decreased until day 16 and slightly increased again until day 21. Apparently, a slightly unstable, dynamic steady state of terpene formation and further processing was established in the stationary phase. Therefore, all subsequent cultivations to evaluate the impact of different food and agro-industrial side-streams on fungal growth and sesquiterpene formation were carried out for a period of eleven days.

### 3.2. Impact of Food and Agro-Industrial Side-Streams

Six side-streams of the food industry were selected as supplements: rapeseed press cake (rapeseed oil production), maple wood (maple syrup production), wheat bran (wheat flour production), potato starch (French fry production), grape pomace (winemaking), and lemon peel (lemon juice production). They were added to the liquid culture medium to induce and increase the biosynthesis of terpenes. Rapeseed press cake, the residue after oil extraction, is a known source of proteins for the feed of dairy animals [41]. It also contains residual 5–18% lipids [42]. Wheat bran and potato starch are both high in carbohydrates and are thought to enhance fungal growth. In 2016, tartrate was used as the medium component for fungal growth [43]. Grape pomace is also rich in oils and phenolic compounds [44] and can be used as a nitrogen source [45]. Citrus peel is rich in flavonoids, d-limonene, and sugars and was already successfully used as a medium supplement [21,46].

**Figure 3:**
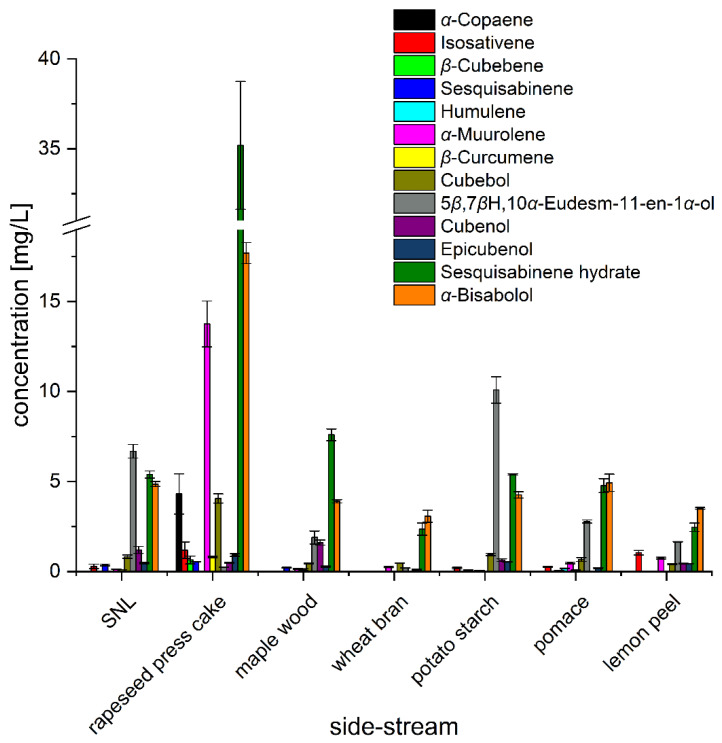
Sesquiterpenoid production during the submerged cultivation of *Cerrena unicolor* for eleven days, supplemented with side-streams from the food and agro-industry.

For all cultures of Cun supplemented with different side-streams, two general statements can be made. First, the course of D-glucose consumption was not noticeably affected (data not shown), and second, no additional sesquiterpenoid products other than those shown in Table 1 were detected. Nonetheless, the side-streams showed a distinct impact on both the overall sesquiterpenoid yields and their quantitative formation profiles (Figure 3). 

**Figure 4:**
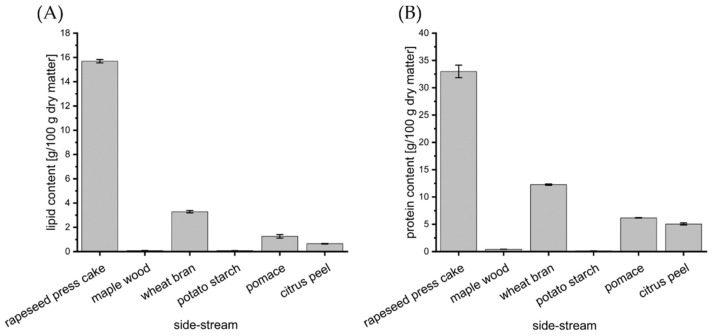
Lipid (**A**) and protein content (**B**) of side-streams used as medium supplements for the cultivation of *Cerrena unicolor*.

Compared to the control (SNL without side-stream), only the addition of rapeseed press cake led to a significantly increased formation of most of the sesquiterpenoids after eleven days of cultivation. *α*-Copaene (4.3 mg/L, not present in control on day eleven), cubebol (4.1 mg/L, 4-fold), sesquisabinene hydrate (35.2 mg/L, 7-fold), *α*-bisabolol (17.7 mg/L, 3-fold), and *α*-muurolene (13.8 mg/L, 100-fold) formation, in particular, were positively affected by the addition of rapeseed press cake. All other terpenoid products experienced only minor changes, compared to the control. The same was true for SNL medium supplemented with the five other side-streams (Figure 3). The main difference in the composition of the side-streams was the high protein (33.0%) and lipid (15.7%) contents of rapeseed press cake in comparison to the other side-streams (Figure 4). A high protein content improved the nitrogen supply of the growing culture and was supposed to increase culture growth and formation of amino acid-derived metabolites, but no changes in fungal growth or immediate metabolites (data not shown) were observed. The following cultivations were thus designed to determine the specific impact of rapeseed press cake on the formation of sesquiterpenoids.

### 3.3. Impact of Rapeseed Press Cake and Lipidic Supplements

The focus was placed on the lipid content and composition. Two possible explanations for the impact of the lipid portion were considered. On one hand, lipid hydrolysis with subsequent *ß*-oxidation of released fatty acids may fill the intracellular acetate pool, which might result in an increased terpene biosynthesis. On the other hand, a lipid phase in the aqueous culture medium may extract lipophilic substances (in situ recovery). This may protect terpenoids, once formed, against catabolism, shift biochemical equilibria towards product formation, and enable high extracellular product accumulation, even above the water solubility of the respective terpenoids.

To characterize the lipid phase of rapeseed press cake, its fatty acid composition was determined. The fatty acid profile is shown in Figure S1. In accordance with literature, rapeseed oil was dominated by unsaturated C_18_ fatty acids, which made up more than 80% (oleic 55%, linoleic 20%, and linolenic acid 8%) [47,48]. Palmitic acid with 13% was the only saturated fatty acid with a higher share in the oil.

To shed a light on the impact of rapeseed oil, single free fatty acids, as well as lipids with a high degree of unsaturated or saturated fatty acids, were chosen as media supplements. Cultivations with Cun in SNL medium were supplemented with (2% *v*/*v*) rapeseed oil, oleic acid, linoleic acid, or coconut butter. The latter is known as a lauric oil rich in lauric, myristic, and palmitic acid and served as counterpart to the highly unsaturated rapeseed oil (Figure S1).

**Figure 5:**
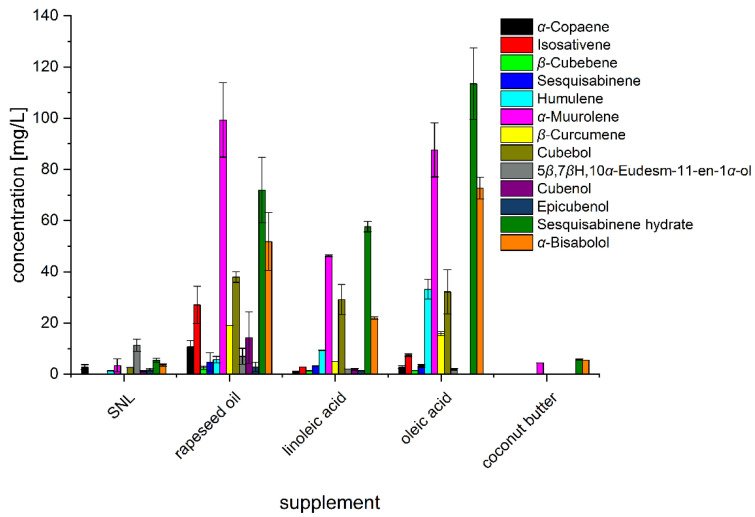
Sesquiterpenoid production of *Cerrena unicolor* supplemented with 2% lipidic additives after eleven days of cultivation. Products were analyzed using TDS-GC-MS after the liquid–liquid extraction of culture supernatant.

As shown in Figure 5, huge differences in terpenoid production were observed bet-ween the control (SNL without supplementation) and coconut butter on the one hand and unsaturated C_18_-fatty acids and rapeseed oil on the other hand. After supplementation with coconut butter, the diversity of the identified terpenoids was strongly reduced. *α*-muurolene, sesquisabinene hydrate, and *α*-bisabolol were the only terpenoids found after eleven days of incubation. Their concentrations were comparable to the respective one in the sample without supplement. Therefore, the meaningful impact of this lipid as the in situ extraction phase was ruled out.

A completely different behavior was observed for the supplementation with oleic acid, linoleic acid, and rapeseed oil. For all three supplements, significantly higher concentrations of sesquiterpenes were produced, compared to both the control (SNL) and the rapeseed press cake (Figure 3). The highest product yields were found for *α*-muurolene and sesquisabinene hydrate in the cases of oleic acid and rapeseed oil supplementation. The yields of both sesquiterpenes were reciprocal, so a purely additive effect of the two fatty acids can be ruled out. The release of fatty acids from the respective triacylglycerols and additional fatty acids, which are less abundant, may have influenced the quantity of particular sesquiterpenes. As rapeseed oil consists of more than 50% oleic acid, the mono unsaturated fatty acid seemed to have the greatest impact on terpene formation. However, as a molecule with an unbranched chain of carbon atoms, neither oleic acid itself nor larger degradation products thereof can serve as direct precursors for sesquiterpene formation.

An active or passive uptake of oleic acid into growing fungal cells and subsequent *β*-oxidation would result in an excess of acetyl-CoA, which may not be exploited completely in the citric acid cycle, especially in the presence of a concurrently active glycolysis. In such situations of excesses of primary metabolites, a switch to secondary metabolism may occur independently from the actual growth phase. Wood-decomposing basidiomycetes are especially known for their highly adaptable metabolism [49,50]. Cun was found in this study to be a producer of cyclic sesquiterpenes (Table 1, Figure 2), which is indicative of an active mevalonate pathway and the expression of several sesquiterpene cyclases [23].

### 3.4. Labeling Experiments

Presuming that a surplus of acetate would trigger the mevalonate pathway, a direct supplementation with ^13^C-labeled acetate should result in an at least partial incorporation into the sesquiterpene/terpenoid products.

After feeding 12 mM ^13^CH_3_COO^-^ on day one, the products were recovered on culture day eleven. The same product spectrum and a terpenoid yield slightly higher than in the control (without acetate supplementation) were obtained. Furthermore, an altered isotopic pattern of the respective molecular ions at *m*/*z* 204 (sesquiterpenes), *m*/*z* 220 (sesquiterpene alcohols), and *m*/*z* 222 (sesquiterpene hydrates) was detected for all of the products. An excess of acetate increased the formation of sesquiterpenes in liquid cultures of Cun (data not shown).

**Figure 6:**
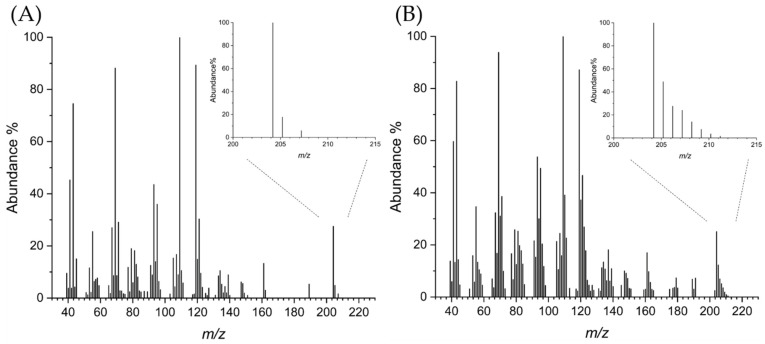
Mass spectra of *α*-bisabolol. (**A**) Spectrum of *α*-bisabolol (scan *m*/*z* from 33 to 300). (**B**) Spectrum of *α*-bisabolol after supplementation of ^13^C-labeled acetate (scan *m*/*z* from 33 to 300). *m*/*z* range from 200 to 215 is shown superimposed in the upper right corner of the respective spectra.

Figure 6 shows exemplarily the electron impact (EI) mass spectrum of the superimposed region around the M-H_2_O ion (the molecular ion at *m*/*z* 222 was not present) of unlabeled (control) and labeled *α*-bisabolol. The control showed the natural ^13^C-isotopic peak at *m*/*z* 205, with 18.3% (measured, 16.5% calculated) intensity of the M-H_2_O ion at *m*/*z* 204. Another peak at *m*/*z* 207 corresponded to the fragment ion after the splitting of a methyl group ([M-CH_3_]^+^) from the molecular radical cation. The isotopic pattern obtained for *α*-bisabolol after feeding ^13^CH_3_COO^-^ was markedly different. There was a number of additional isotopic peaks after the [M-H_2_O]^+^ ion from *m*/*z* 205 to *m*/*z* 211, ordered in a falling ladder of intensity. This can be explained by the incorporation of up to six ^13^C-labeled acetates (one ^13^C per acetate). Further expected isotopic peaks were missing, due to their low abundance, because only a distinguished share (roughly 30% (calculated with the percental abundance of *m*/*z* 205 and 206, Table S2)) of labeled acetate besides the ubiquitous unlabeled acetate built up the *α*-bisabolol molecule. The regular isotopic distribution of multiple-labeled *α*-bisabolol at *m*/*z* 204 was altered from *m*/*z* 207 because of the contribution of the [M-CH_3_]^+^- fragment ion, which made further comparisons of measured and calculated isotopic patterns complicated. Therefore, the incorporation of labeled acetate in all sesquiterpenoids produced by Cun was verified *via* the increased abundance of *m*/*z* 205 and the occurrence of *m*/*z* 206 (Table S2) only.

Humulene was the only sesquiterpene with an unusually high intensity (39.5%) of the isotopic peak at *m*/*z* 205 already in the control. A co-elution with another compound was most likely parental for this, but the incorporation of labeled acetate was clearly indicated by the occurrence of *m*/*z* 206, which was not present in the control.

For low-yield products, not all of the expected labels were detected, due to lower ion intensities. Nevertheless, the incorporation of ^13^C-labeled acetate was detected for all sesquiterpenoids produced by Cun and verified that a surplus of acetyl-CoA had flown into the mevalonate pathway. It is noteworthy that predominately cyclic sesquiterpenoids were generated. The constant development of genome mining and bioinformatic tools in general led to an increasing number of characterized sesquiterpene biosynthetic pathways [51]. In a previous work, ten cyclases were identified in Cun and heterologously produced [23]. This finding is in good accordance with the results of the present study.

## 4. Conclusions

*Cerrena unicolor* possesses a unique portfolio of sesquiterpene cyclases, which enables the biotechnological production of a number of bioactive mono- and bicyclic sesquiterpenes (Table 1). Their production was boosted to a total of more than 300 mg/L by feeding them unsaturated fatty acids, which exceeded the solubility of some sesquiterpene hydrocarbons in water. For example, the predicted water solubility of *α*-muurolene (ALOGPS solubility calculation software) was 10 mg/L (https://foodb.ca/compounds/FDB016928, accessed on 20 December 2022), but the almost 10-fold concentration was isolated from a culture broth upon supplementation with rapeseed press cake (Figure 3). Residual lipid droplets in the culture supernatant may have served as protective accumulation sites, which would explain the high product concentrations observed. Rapeseed press cake was a sustainable nutrient source to improve sesquiterpenoid production because it is a large-volume, lipid- and protein-rich side-stream of rapeseed oil production (Figure 4). In this study, its double function as a precursor and as an accumulation site was supposed.

Knowledge about the biogenesis of sesquiterpenoids and its regulation in basidiomycetes is deficient [52]. An overflow of the acetyl-CoA pool appeared to stimulate the mevalonate pathway. This hypothesis was strongly supported through the incorporation of labeled acetate into the carbon chain of the sesquiterpenes. Further improvements in product yields can be envisaged through a systematic optimization of cultivation conditions. However, substantial improvements may be achieved through strain engineering only. Unfortunately, only very few data on the regulation of gene expression and no data on terpene metabolism of Cun on the genome level are available [53]. Experimental tools to modify the genome of Basidiomycota are still under development and need to be refined in the future [54].

Biotechnologically generated sesquiterpenes used as flavor compounds may be labeled ‘’natural,’’ according to current EU regulations (VO (EG) 1334/2008). In the United States, natural flavors are defined under regulation 21 CFR 101.22. Under this regulation, “natural flavor” or “natural flavoring” means “…. any product of …. plant material …. or fermentation products thereof, whose significant function in food is flavoring ….”. As naturalness of food is crucial for the majority of consumers, this distinguishing attribute creates a marketing advantage for the producer [55].

## Figures and Tables

**Table 1:** Volatile sesquiterpenoids produced by *Cerrena unicolor*.

Clade	Substance	RI Polar ^a (b)^	RI Non-Polar ^a (b)^	Identification
II	*α*-Copaene	1462 (1502)	1349 (1375)	A, B, C, E
I	Isosativene	1492	1390 (1417)	B, C
I	*β*-Cubebene	1544 (1540)	1390 (1388)	A, B, C, E
III	Sesquisabinene	1663 (1648)	1454 (1465)	A, B, C, E
IV	Humulene	1693 (1670)	1460 (1455)	A, B, C, E
II	*α*-Muurolene	1729 (1727)	1501 (1499)	A, B, C, E
III	*β*-Curcumene	1743 (1734)	1510 (1516)	A, B, C
II	Cubebol	1956 (1951)	1521 (1531)	A, B, C, E
II	5*β*,7*β*H,10*α*-Eudesm-11-en-1*α*-ol	2001	1544	C
II	Cubenol	2074 (2063)	1641 (1642)	A, B, C, E
II	Epicubenol	2083 (2072)	1635 (1625)	A, B, C
III	Sesquisabinene hydrate	2114 (2092)	1596 (1581)	A, B, C
III	*α*-Bisabolol	2223 (2232)	1694 (1697)	A, B, C, D, E

^a^ Retention index; ^b^ literature retention index (NIST data base: https://webbook.nist.gov/chemistry (accessed on 21th October 2022)). A/B identified *via* retention index (polar/non-polar column); C identified *via* comparison of MS spectra (NIST, Wiley spectral database); D identified *via* authentic standard; E identified *via* odor impressions obtained by GC-O.

## Data Availability

Data are contained within this article and Supplementary Materials.

## Supplementary Materials

The following are available online at https://www.mdpi.com/article/10.3390/foods12030668/s1; Table S1: Odor impressions obtained by GC-O measurement of extracts from cultivation of Cerrena unicolor in SNL and rapeseed oil as media supplement; Table S2: Intensities of sesquiterpenoid molecular ion (*m*/*z* 204) and isotopic peaks (*m*/*z* 205, *m*/*z* 206) with and without supplementation of ^13^C-sodium acetate.
