# Supplementary material for: Impact of Agro-Industrial Side-Streams on Sesquiterpene Production by Submerged Cultured Cerrena unicolor
Source: Foods. 2023 Feb 3;12(3):668. doi: 10.3390/foods12030668 (PMC9914794; doi:10.3390/foods12030668)
Supplement: Supplementary file 1 [file foods-12-00668-s001.zip › foods-2142449-supplementary.pdf]

Table S1: Odor impressions obtained by GC-O measurement of extracts from cultivation of *Cerrena unicolor* in SNL and rapeseed oil as media supplement.

peak	substance	odor impression
1	α-Copaene	fresh, honey
2	Isosativene	
3	β-Cubebene	citrus, unpleasant
4	Sesquisabinene	woody
5	Humulene	woody
6	α-Muurolene	woody
7	β-Curcumene	
8	Cubebol	spicy
9	5β,7βH,10α-Eudesm-11-en-1α-ol	
10	Cubenol	heavy, tea
11	Epicubenol	
12	Sesquisabinene hydrate	
13	α-Bisabolol	flowery

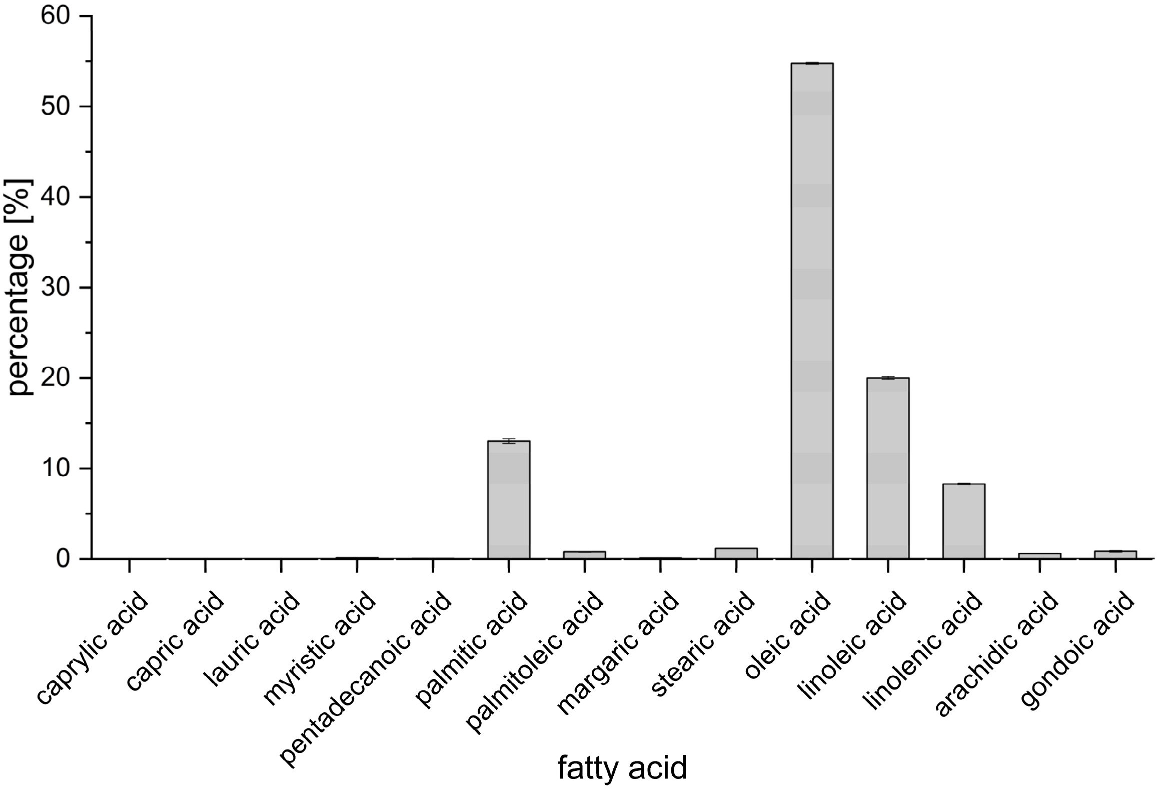

Figure S1: Fatty acid distribution of residual oil from rapeseed press cake obtained *via* Soxhlet extraction.

Table S2: Intensities of the sesquiterpenoid molecular ion (204 m/z) and isotopic peaks (205 m/z, 206 m/z) in the supernatant after eleven days of cultivation of *Cerrena unicolor* in SNL w/wo supplementation of ^{13}C sodium acetate.

m/z	α-Copaene		Isosativene		β-Cubebene	
	SNL control	^{13}C-acetate	SNL control	^{13}C-acetate	SNL control	^{13}C-acetate
204	100	100	100	100	100	100
205	16.0	29.3	16.8	22.5	15.3	28.5
206		4.7		3.5		4.6

m/z	Sesquisabinene		Humulene		β-Curcumene	
	SNL control	^{13}C-acetate	SNL control	^{13}C-acetate	SNL control	^{13}C-acetate
204	100	100	100	100	100	100
205	18.2	30.4	39.5	46.5	21.8	30.9
206		5.1		18.4		6.2

m/z	α-Muurolene		Cubebol		5β,7βH,10α-Eudesm-11-en-1α-ol	
	SNL control	^{13}C-acetate	SNL control	^{13}C-acetate	SNL control	^{13}C-acetate
204	100	100	100	100	100	100
205	18.6	37.5	20.2	29.7	17.0	31.9
206		10.9		5.3		6.5

m/z	Cubenol		Epicubenol		Sesquisabinenhydrat	
	SNL control	^{13}C-acetate	SNL control	^{13}C-acetate	SNL control	^{13}C-acetate
204	100	100	100	100	100	100
205	28.8	46.6	23.1	38.6	16.6	69.5
206		8.0		6.2		15.9

m/z	α-Bisabolol	
	SNL control	^{13}C-acetate
204	100	100
205	18.4	48.8
206		27.5
